# Lectures Illustrative of Certain Local Nervous Affections

**Published:** 1837-07

**Authors:** 


### Art. VIII.

*Lectures illustrative of certain Local Nervous Affections.* By Sir Benjamin C. Brodie, Bart., F.R.S., Serjeant Surgeon to the King, and Surgeon to St. George's Hospital.—*London*, 1837. 8vo. pp. 88.

Nothing is more true than that there is a great deal of practical knowledge, which experience only teaches, which systems of medicine and surgery do not comprehend, and which is only to be communicated by commentaries on certain orders of symptoms, "which, while they have many characters in common, may arise from various causes, and require very different modes of treatment for their relief." It is for the purpose of communicating knowledge of this kind that Sir B. Brodie publishes these lectures, which are full of practical information, and cannot but be gratefully received from so eminent a surgeon by the profession at large.

The first words of Sir Benjamin's first lecture will, we imagine, remind many a practitioner of the troubles of those undefinable and intractable cases which few can have been so fortunate as not to meet with, and one of the griefs of which has been "a constant and severe pain referred to a spot, about three or four inches in diameter, in the situation of the false ribs of the left side." It may be instructive, or at least consolatory, to know, that in this particular case, the subject of which was hysterical, examination after death detected no morbid appearances in the affected part; a fact which will perhaps be admitted as a sufficient explanation why the repeated application of leeches, very diligent blistering, and other ingenious local measures so often fail to give relief.

In cases like these, observes Sir B. Brodie, the nerves of sensation, as in others the nerves of motion, are morbidly affected; in the one case pain, and in the other spasm, or total loss of power, is the effect; and the question for the practitioner to solve is, where does the irritation of the nerve commence? The instance of a man is mentioned who complained of severe pain in the inside of his knee, which was relieved when the femoral artery was tied for the cure of an aneurism in the thigh, some branches of the crural nerve passing over the aneurismal tumour, and kept on the stretch by it, terminating in the part to which the pain had been referred. In disease of the hip, the surgeon well knows how often pain is referred to the knee; and the physician often meets with very acute pains affecting the legs in cases of phthisis, in which the nervous irritation seems at first sight remote from any presumable cause. Such pains are not always to be explained by the common origin of affected nerves, any more than the pain in the right shoulder so commonly attending disease of the liver, or the pain in the back in some cases of disease of the heart. Acid in the stomach has not unfrequently been accused of exciting pains in the legs and feet. A pint of claret ensures the gout to some offenders in a few hours; and we have met with individuals in whom the cool refreshment of hock in summer could not be enjoyed without the disadvantage of many wandering pains the following day. Sir Benjamin quotes a case from Dr. Denmark, in the Medico-Chirurgical Transactions, of a sailor who " received a wound from a musket ball in the arm: the wound healed, but the patient complained of an agonising pain, beginning in the extremity of the thumb and fingers (except the little one), and extending up the forearm. His sufferings were such that he willingly submitted to the amputation of the limb, and the operation gave him complete relief. On dissecting the amputated limb, a small portion of lead, which seemed to have been detached from the ball when it had struck against the bone, was found imbedded in the fibres of the median nerve." (P. 6.)

Similar effects, it is observed, are produced where the actual seat of the disease is in the brain or the spinal marrow. Of this instances must, we think, often have been remarked in cases of paralysis, as well as in epilepsy; and they have also been witnessed, without being understood, in cases pronounced to be either anomalous or feigned; particularly where the seat of the pain was occasionally shifted. In cases of an analogous kind, neuralgic pains alternate with insanity.

Among the examples given by Sir B. Brodie of pains arising from some distant local irritation, is a remarkable one (p. 12,) of pain in the foot produced by stricture of the urethra, and relieved by the use of a bougie. We believe that Dr. Sanders of Edinburgh ascribed many cases of gout to the same cause, and, in his lectures, held them to be curable in the same way. Sir Benjamin mentions another case, in which the pain of the ankle and toes was connected with hemorrhoids, and cured by lavements of cold water and the use of Ward's paste (*confectio piperis composita*) taken three times daily, and lenitive electuary at bed-time.

" Now, in such cases as these, you will at once perceive that there is no direct communication between the nerves of the parts affected that will afford a reasonable explanation of the occurrence of the sympathetic pain; and you will naturally enquire, how then is the sympathetic pain produced? To this question I would answer, that in all probability it is in the brain itself that the communication is made, the impression being first transmitted to the sensorium, and from thence reflected to the nerves of the part which is secondarily affected. If you dissect the brain according to Reil's method, having first hardened it by maceration in alcohol, you will find it splitting into fibres, passing in various directions, many of which may be demonstrated as connecting even the most distant convolutions of the cerebrum with each other; and if, with the limited knowledge which we at present possess, we venture to speculate on this obscure but interesting subject, we may easily be led to suppose that an impression on one part of the body should, by means of these communicating fibres, produce a disordered sensation in another part. It is not more improbable that this should happen than it is that the whole fabric of the nervous system should sympathize with an affection of a particular nerve, as is the case in traumatic tetanus, and on many other occasions of which the experience of surgeons will furnish numerous instances." (P. 14.)

A remarkable feature of these affections, it is observed, is that they seem to be suspended during sleep; not only nervous pains, but nervous spasms. They have also marked intermissions and exacerbations, to which the name of spasm is sometimes erroneously applied even to characterize the pains. The nervous pains are to be distinguished, Sir Benjamin Brodie says, from those produced by inflammation "by the absence of throbbing, by their not being increased by pressure, by there being no evident turgescence of the small vessels;" but he acknowledges that there is much difficulty in the diagnosis when the affection has lasted some time; that increased vascularity may follow, and swelling, and actual inflammation. Where the intermissions are as regular as those of an ague, the quinine gives no less relief than in ague. In other cases the pain may depend on disorder of the bowels or on actual disease of the brain, without any particular difference being observable in the symptoms. The following passages comprehend some important remarks on the forms of such affections, or the parts they most commonly appear in.

"Although there is no part of the body which may not, at one time or another, be the seat of these nervous affections, it would appear that some parts are more liable to them than others. They are met with less frequently in the viscera, which are supplied by the great sympathetic nerves, than in other parts. Nervous pains are more severe, and perhaps, on the whole, more common, in those parts which receive their nerves from the fifth pair, as the face, the eye, the tongue, than in any other individual part. Muscular spasms are common in the muscles of the neck, especially in the sterno-cleido-mastoideus. I am inclined to believe, also, that they occur more frequently in the upper limb than in the lower. It is not uncommon to see one hand and arm in a state of constant tremulous motion, there being no other indication of disease. I have seen several cases in which a muscular spasm of the upper limb has shown itself in the following manner. The patient experiences no inconvenience from it until he uses the limb; for example, until he sits down to write. Then, when he has gone so far as to have written a few letters, some of the muscles act involuntarily, and jerk the hand in a direction contrary to that which was intended; so that, instead of completing the word which was begun, the pen makes a long scratch on the paper.

"A lady complained of pain in her head, and her mouth was drawn to one side, and hence she was supposed to suffer from paralysis of the muscles of one side of the face. However, when I was consulted respecting her, I observed that there were nearly constant twitches of the cheek and eyelids on that side to which the mouth was drawn; and, on more minute examination, I was satisfied that the distortion of the mouth arose not from the muscles on one side of the face being paralytic, but from those on the opposite side being in a state of spasm. The case precisely resembled that of a patient with spasmodic wry neck, except that the disease influenced a different set of muscles, namely, those supplied by the facial nerve, or the *portio dura* of the seventh pair.

"Perhaps there are no muscles in the body which are, on the whole, more liable to have their actions deranged under the influence of nervous disorders than those of the pharynx and œsophagus. In not a few of these cases, which have been confounded together under the general appellation of stricture of the œsophagus, the disease is either a spasmodic or a partial paralytic affection of these parts, and the patient is to be cured, not by the introduction of bougies into the œsophagus, but by other means." (P. 20.)

Cases of this kind are not only curious as studies, but baffling as regards treatment. Sir Benjamin reprobates the indolent and careless application of specifics, given in the hope that something may hit the disease; and recommends that each case should be studied pathologically, so that the symptoms may be traced to their real origin. His illustrations are so instructive that we cannot but quote them:—

"A patient applies to you complaining of a pain in the testicle, but the testicle appears to have its natural structure, and (except the pain) bears no marks of inflammation. You enquire further, and find that the pain is not constant; that it is especially induced by exercise, and that it subsides when the patient is in the horizontal posture. Examine the groin after he has taken a long walk, and you will perhaps find an incipient hernia; a small portion of bowel just attempting to protrude through the abdominal ring. You apply a truss, which supports the hernia, and cures the pain in the testicle. If you had been careless in your investigation of the case, and had applied leeches and lotions to the testicle, you would, to say the least, have plagued your patient to no purpose.—Another person applies to you concerning a spasmodic wry neck. If you at once conclude that the disease is where it shows itself, and divide the tendon of the sterno-cleido-mastoideus muscle, what is the consequence? The patient undergoes a certain quantity of pain in the operation, and to no purpose; for, before the wound is completely cicatrized, the divided tendon has again become fixed by adhesion to the neighbouring textures, and the contraction of the muscle and the twisting of the neck are as bad as ever. I shall relate a case in which a patient underwent a severe and painful operation to no purpose, in consequence of such a want of discrimination on the part of the surgeon. A sailor had received a severe wound in the ham, I believe from a musket-ball. The wound healed, but not until after a considerable time, and the patient was left with a contracted leg, and suffering from a most agonizing pain in the foot. This state of things having existed for a considerable time, and no benefit having been derived from any of the remedies employed, the poor fellow wished to lose the foot. The surgeon under whose care he was, therefore, amputated the leg. But, unfortunately, he amputated it, not above the knee and above the injury of the nerve, but below the knee and below the injury. I scarcely need tell you the result. The pain continued as severe as ever, and it was not relieved until amputation had been performed a second time higher up in the limb." (P. 24.)

The author's remarks on those cases which depend on irritation of the digestive mucous membrane, or which are connected with gout, or with intermitting fever, are very interesting; of the latter, some extremely striking cases are given. When the cause of the local nervous affection is organic disease of the brain, Sir Benjamin recommends local applications of hemlock, belladonna, stimulating liniments with laudanum, and even blisters, for the palliation of pain.

The Second Lecture in this little work relates to the Various Forms of Local Hysterical Affections, and is full of curious particulars. One of the most remarkable of these, and perhaps one of the most important to the practitioner, is that in which a *joint* is affected with pain connected with hysterical phenomena, and in which the pain and morbid sensibility may lead the practitioner to suppose that the joint is diseased. The frequency of such a form of affection in women of the higher classes is indeed astonishing. "I do not hesitate to declare," (says Sir B. Brodie,) "that among the higher classes of society, at least four-fifths of the female patients, who are commonly supposed to labour under diseases of the joints, labour under hysteria, and nothing else." (P. 37.) We strongly recommend every enterprising young surgeon to read Sir Benjamin's observations on the diagnosis of these cases, the result of a large experience, and conveyed in simple and intelligible language. Such an affection may originate in a severe illness, or arise from some moral cause having a depressing influence on the constitution. The symptoms generally come on gradually, last long, and gradually subside; but sometimes they yield suddenly.

In other cases, the spine is the seat of the hysterical affection, and disease of the cartilages or the vertebræ being erroneously supposed to exist, the horizontal posture, and perhaps caustic issues and setons, are resorted to, in the case of unfortunate young ladies, "for several successive years, in whom air and exercise, and cheerful occupations, would probably have produced a cure in the course of a few months." (P. 47.) All Sir Benjamin's observations on these cases, also, merit the reader's best attention. With respect to the paralysis that often accompanies them, he observes of this peculiarity, that "it is not that the muscles are incapable of obeying the act of volition, but that the function of volition is not exercised." (P. 48.) He extends this observation to cases of hysterical retention of urine, at least in the first instance; and the following practical remark is valuable:—

"Females who labour under hysterical retention of urine, if left to themselves, usually recover in the course of a short space of time, sometimes almost suddenly; but if the catheter be employed, their recovery may be protracted for an indefinite period. We may lay it down as a general rule, that in these cases the catheter should not be had recourse to: and the only exceptions to it are in those extreme cases in which actual paralysis has taken place, and the bladder is likely to become diseased if not artificially relieved." (P. 50.)

Several useful practical remarks occur in the pages devoted to hysterical aphonia, hysterical tympanitis, hysterical affection of the breast, and the severe forms of hysterical affection sometimes supervening on slight accidental injuries; to all of which we can only strenuously recommend the student's careful attention.

The Third and concluding Lecture is occupied with the Pathology of Hysteria, and the Treatment of Local Hysterical Affections. Three very convincing cases are related, in which, after severe and varied hysterical affections, death ensued, and the most careful examination could detect no alteration in the state of the brain, spinal cord, or other parts of the body, except in the latter (as in the bladder or intestines,) such as were produced by secondary affections; but he justly observes that constitutional differences in the organization of the nervous system may exist which are not palpable to the senses. The same apparent absence of disease is well known to exist in some cases where the mind has been violently affected. Sir Benjamin thinks that in the generality of hysterical cases, "there is an evident weakness and laxity of the tissues, independently of what may be supposed to belong to the tissues of the nervous system." It cannot be doubted that they often exhibited a great want of physical power, and many marks of a feeble circulation, and of a deficiency of nervous energy.

The observations of Sir Benjamin Brodie on the Treatment of these affections is, as may be supposed, most judicious. He forcibly points out the evils committed in the conduct of early education, and exhorts his hearers to point out to the more affluent classes of society the errors into which they fall in this respect. In the general treatment, he observes that the whole class of tonics may, under certain circumstances, be employed with advantage; and that the patient should live on a generous diet, should take exercise out of doors, live in the country, and have the mind agreeably occupied. Among tonics, Sir Benjamin ranks the valerian and assafœtida; and he speaks highly of the sulphate of copper in small doses. In particular cases, the state of the bowels, or of the uterus, demands especial attention; and "it is not unusual in aggravated cases of hysteria to find the urine depositing a large quantity of lithic acid, in the form of sand; or the urine may be voided high-coloured, depositing a pink amorphous sediment, abounding in the lithate of ammonia; and in either of these cases the exhibition of alkalies, combined with alterative doses of mercury, purgatives, and a regulated diet, will contribute to produce a cure; the unhealthy quality of the urine seeming to be the cause, rather than the effect, of the hysterical affection." (P. 76.)

We have already mentioned the local applications recommended for the relief of the pain of some of the local hysterical affections. Although the loss of blood sometimes gives relief, repeated bloodlettings are highly injurious to the patient, generally, in Sir Benjamin's experience, causing the patients to become invalids for life; but the patient's attachment to frequent abstractions of blood is often very great, in consequence of the temporary relief thus obtained. Deprecating, as we do, general bloodletting in such cases, as also the application of leeches to the pained part, we must admit that the latter remedy is often extremely beneficial in that class of nervous pains which seem traceable to some unknown condition of the spinal marrow, commonly termed *spinal irritation*. In these cases there often exists local tenderness on the spine, and the application of a few leeches to this part is frequently followed by immediate relief of the pain at a distance. Blisters, issues, and other counter-irritants, applied to the seat of the pain, are generally worse than useless; and whatever treatment is adopted should "involve as little as possible deviation from the ordinary habits of life." In the case of hysterical neuralgia of the knee or hip, amendment seldom takes place until the patient is induced, notwithstanding present suffering, to use the limb. Sir B. Brodie's experience is against the attempt to effect a cure from the division of the nerves supplying the affected part.

In conclusion, the accomplished surgeon whose remarks we have done little more than follow throughout this notice, makes some very useful reflections on the occasional disappearance of hysterical symptoms without any manifest cause for their disappearance; or, more frequently, after some forcible impression made on the nervous system; circumstances which explain some apparent miracles effected by saintly men and by popular quacks: and, as he has warned his pupils against mistaking cases of nervous affection for real local disease, he does not fail to caution them against overlooking disease when it really exists.

